# Draft Genome Sequences of Four Species of *Chlamydomonas* Containing Phosphatidylcholine

**DOI:** 10.1128/genomeA.01070-16

**Published:** 2016-09-29

**Authors:** Takashi Hirashima, Naoyuki Tajima, Naoki Sato

**Affiliations:** a Department of Life Sciences, Graduate School of Arts and Sciences, University of Tokyo, Tokyo, Japan; b Core Research for Evolutional Science and Technology, Japan Science and Technology Agency, Tokyo, Japan

## Abstract

Phosphatidylcholine (PC) is one of the essential phospholipids for most eukaryotes. Although the model green alga *Chlamydomonas reinhardtii* lacks PC, four species containing PC were found in the genus *Chlamydomonas*. Here, we report the draft genome sequences of the four species of *Chlamydomonas* containing PC.

## GENOME ANNOUNCEMENT

Most eukaryotic organisms contain phosphatidylcholine (PC), but various algae, including a unicellular green alga, *Chlamydomonas reinhardtii*, are known to lack PC (1, 2). In these algae, the phosphorus-free betaine lipid diacylglyceryl-*N*,*N*,*N*-trimethylhomoserine (DGTS) has been thought to function in place of PC (1, 2). In some nonphotosynthetic microorganisms possessing both PC and DGTS, phosphate starvation induced a decrease in PC and an increase in DGTS (3, 4). The apparent complete replacement of PC with DGTS in PC-lacking algae could be a result of adaptation to a phosphorus-limited environment.

We recently detected PC in four species of the genus *Chlamydomonas*: *C. applanata* NIES-2202, *C. asymmetrica* NIES-2207, *C. debaryana* NIES-2212, and *C. sphaeroides* NIES-2242 (5). PC biosynthetic pathways and the enzymes involved therein have been revealed in yeasts, mammals, and land plants (6) but remain unclear in most algae.

*C. applanata* NIES-2202, *C. asymmetrica* NIES-2207, *C. debaryana* NIES-2212, and *C. sphaeroides* NIES-2242, which were obtained from the Microbial Culture Collection at the National Institute for Environmental Studies, Japan, were grown photoautotrophically in modified Bristol’s medium (7). Genomic DNA from each of these species was released by treatment with proteinase K and sodium *N*-dodecanoylsarcosinate and isolated by CsCl density gradient ultracentrifugation, as described previously (8). Purified DNA was submitted to paired-end sequencing by Illumina HiSeq 2000 (*C. sphaeroides*) or MiSeq (other three species) through the sequencing service of TaKaRa Bio, Inc. (Otsu, Japan). The obtained reads were assembled using the software Velvet version 1.2.08 (9).

The total length of draft genomes in *C. sphaeroides* and *C. debaryana* that are closely related to *C. reinhardtii* (10) was also close to the genome size of *C. reinhardtii* (around 120 Mbp) (11). The other two species had genomes that were smaller (79 Mbp, *C. applanata*) or larger (145 Mbp, *C. asymmetrica*) than that of *C. reinhardtii*, showing considerable variation in genome size within the genus *Chlamydomonas*.

Putative genes involved in the biosynthesis of PC were searched using the tblastn program (12). The three-step methylations of phosphatidylethanolamine and/or phosphoethanolamine are necessary for the *de novo* synthesis of PC, and they are catalyzed by phosphatidylethanolamine-*N*-methyltransferase (PEMT) and/or phosphoethanolamine-*N*-methyltransferase (PEAMT), respectively. All four species analyzed in the present study were found to harbor a single putative gene coding for PEMT, whereas a putative gene encoding PEAMT was found in *C. applanata* and *C. asymmetrica* only. These results suggest that at least two different types of pathways exist for the PC biosynthesis in these species. The draft genome sequences reported here, however, will be useful in finding not only lipid-related genes (13) but also genes involved in diverse cellular functions.

### Accession number(s).

The draft genome sequences of the four *Chlamydomonas* species were deposited in DDBJ/EMBL/GenBank under the accession numbers listed in Table 1. The version described in this paper is the first version.

**TABLE 1:** Genome features and GenBank accession numbers of sequenced species

Species	Accession no.	Approximate genome size (Mbp)	No. of scaffolds (>1,000 bp)	Coverage (×)
*C. applanata* NIES-2202	BDCZ00000000	79	2,533	21.4
*C. asymmetrica* NIES-2207	BDDA00000000	145	4,102	11.2
*C. debaryana* NIES-2212	BDDB00000000	126	10,139	10.5
*C. sphaeroides* NIES-2242	BDDC00000000	127	6,890	34.1
